# The generational happiness switch in Norway: the puzzle of miserable youth and happy seniors

**DOI:** 10.3389/fpsyg.2025.1706473

**Published:** 2026-01-07

**Authors:** Nina Witoszek, Mads Larsen

**Affiliations:** 1 Department of Journalism and New Media, Civitas University, Warsaw, Poland; 2 Department of Psychology, Norwegian University of Science and Technology, Trondheim, Norway

**Keywords:** cultural crisis, democratic resilience, senior well-being, social media, youth ill-being

## Abstract

An alarming rise in youth ill-being is sweeping across many parts of the world. In Norway, surveys conducted since 1985 consistently identified young people as the happiest age group, while seniors ranked lowest in terms of well-being. Since the early 2010s, however, this pattern has reversed: happiness among seniors has increased, whereas the well-being of young people has declined sharply. Quantitative research attributes this shift to strained social relationships, mental health challenges, and growing anxiety about the future. Social media appears to exacerbate these difficulties, though its interaction with broader cultural and societal transformations remains poorly understood. This study combines qualitative interviews with Norwegian high school students, retirees, teachers, health professionals, and other youth experts (*n* = 48), alongside an analysis of public debates concerning the causes and manifestations of adolescent distress. We explore four central questions: Is Norway—one of the world’s wealthiest nations—living proof that “money cannot buy happiness”? What are the main causes of youth ill-being? How do young and old Norwegians interpret the new, digitally driven reality? And what explains seniors’ unexpected resilience and well-being amid climate anxiety, geopolitical instability, and economic uncertainty? Our findings suggest that the rise in youth unhappiness—while partly associated with social media use—must be understood within a broader cultural framework: as a symptom of weakening communal bonds and a deepening crisis of the cooperative, prosocial ethos that has long sustained Norwegian society. An open question remains as to the extent to which this young, increasingly competitive and self-centred generation may challenge that ethos and, in a long run, undermine the very foundations of Norwegian social democracy.

## 1 Introduction

From an evolutionary perspective, happiness can be understood as a reward mechanism that signals success in solving adaptively relevant challenges. When individuals find a partner, achieve social or professional success, or exceed personal or social expectations, feelings of happiness indicate that their strategies are effective. Conversely, unhappiness serves as a warning that one’s prospects for survival, reproduction, or long-term well-being may be compromised (Buss, 2000; Hill and Buss, 2008; Larsen et al., 2023). This framework helps explain why younger people have historically reported higher levels of happiness in surveys than older populations. Early adulthood is a life stage defined by key adaptive tasks related to education, work, social relationships, and family formation. Successfully navigating these challenges produces transient or enduring experiences of happiness, which in turn motivate continued effort to climb the ladder of happiness.

Most seniors are no longer engaged in the same breadth of life-defining struggles that characterize youth and early adulthood. While their time, energy, and resources may contribute to the adaptive success of children and grandchildren, individual achievement is typically less central to their well-being. Rather than experiencing intense emotional highs, older adults often derive a sense of *satisfaction*—a form of cognitive well-being rooted in a reflective, holistic assessment of one’s life trajectory (Røysamb and Nes, 2016). In well-to-do countries, age-related illness may hamper physical well-being, but generous welfare, long experience in dealing with challenges, and voluntary work may contribute to how many seniors still report a high quality of life (Hansen and Daatland, 2016; Adhikari and Uddin, 2023).

**Figure 1:**
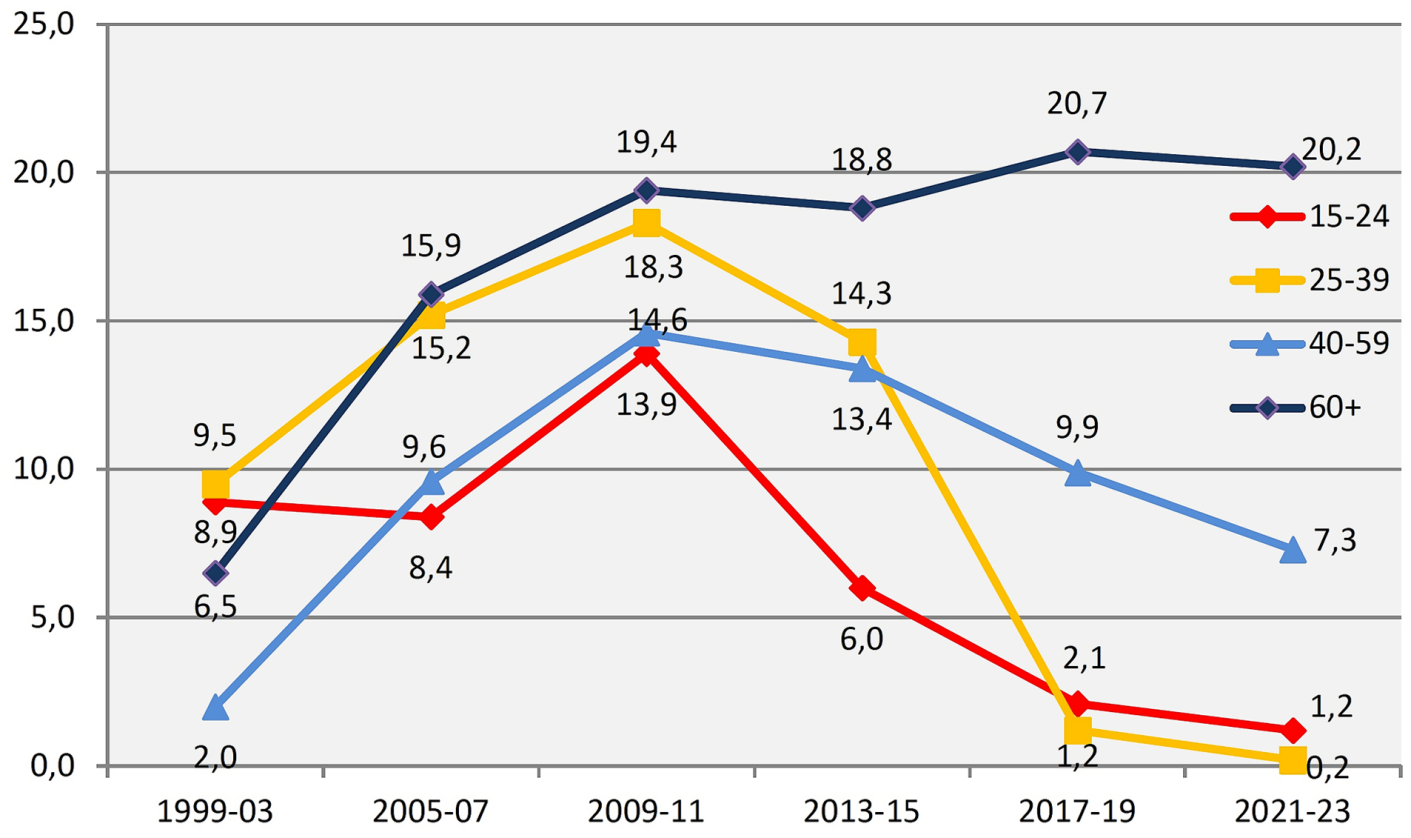
Trends for satisfaction with own life within age groups (percent balance “very satisfied” minus “very or a little dissatisfied or neither satisfied or dissatisfied”). The X axis contains two and two waves combined except for first three. The Y axis is Norwegian Monitor’s satisfaction balance measure.

**Figure 2:**
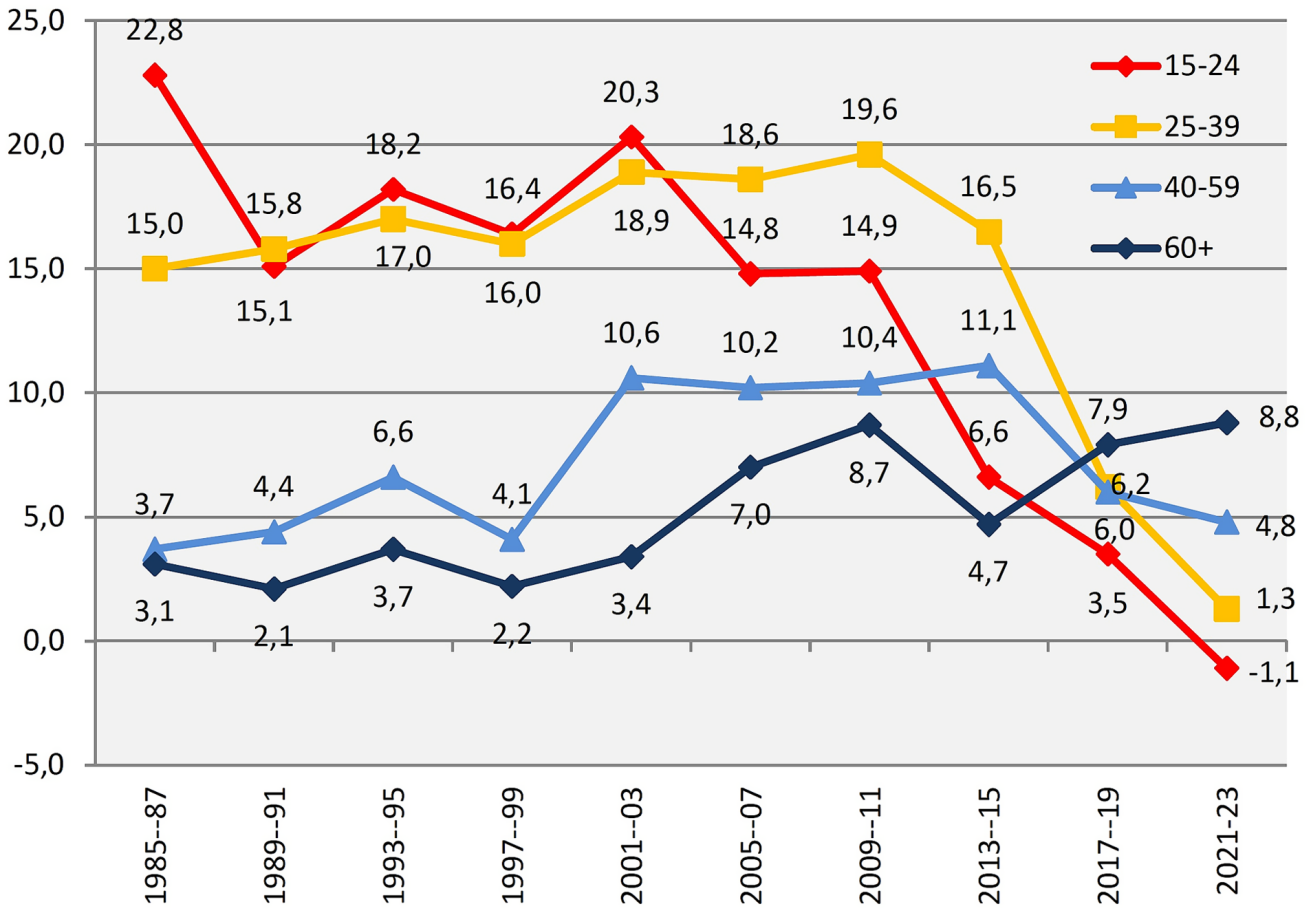
Trends for feeling of happiness within age groups (percent balance “very happy” minus “not particularly or not at all happy”). The X axis contains two and two waves combined. The Y axis is Norwegian Monitor’s happiness balance measure.

Findings from Norwegian well-being surveys reinforce the distinction between happiness and satisfaction (Hellevik, 2008). The *Norwegian Monitor* has conducted biennial, repeated, non-overlapping surveys since 1985, including questions about happiness and, from 1999, satisfaction. Results, illustrated in Figures 1, 2, are reported as balance measures, calculated by subtracting the proportion of neutral or negative responses from the proportion of very positive responses, making them a composite indicator of overall movement in subjective well-being.

In these surveys, those older than 60 have mostly been the cohort with the highest level of *satisfaction* (Figure 1), but were the least happy until 2015 (Figure 2). Respondents in the age group 15–24 showed the highest or second-highest happiness level until 2011. Hellevik (2017), who was in charge of the surveys, summed up the field’s conventional wisdom: “Happiness peaks in the late 20s to early 30s, in the period after having found a partner to live with and before the birth of children. Thereafter, the tendency is for the happiness level to be lower the higher the age of a group, with the exception of a modest increase for those above 70”.^1^ The slight increase in well-being for the very oldest, he surmised, was likely due to “a tendency for happy persons to live longer, and to be more able or willing to participate in a survey than are elderly people generally.”

These assumptions held true for several decades (Larsen and Witoszek, 2024). Exceptional growth and optimism characterized much of the Western world throughout the post–World War II era. However, research indicates that following the 2008 economic downturn and the rapid expansion of social media, a series of economic and cultural transformations have profoundly affected the younger generation. Rising property prices, mounting pressure from educational and professional competition, and widespread anxieties about the future have all been cited as contributing to the pervasive sense of doom and gloom among young people (Foa and Mounk, 2019).

It is perhaps not surprising that the multiple challenges of our time contribute to a generational happiness switch. Again, when seen through an evolutionary lens, ill-being signals to the young that their future is imperiled and that they should strategize accordingly. The situation is different for Norwegian seniors. It is reasonable to presume that, after having lived through an extraordinary boom related to Norway’s growing status as a dizzyingly rich petrostate, they can now harvest the joys of generous, oil-fund-backed pensions, and revel in satisfaction stemming from a fulfilled life.

The elderlies’ experience of happiness and satisfaction—two indicators of well-being that are inherently relative—is likely to increase as people tend to assess their own fortune by comparing themselves to others (Frank, 1999; Hill and Buss, 2006). Seniors who compare their predicament with the prospects of younger generations may feel better about both the life they have lived and their current situation. They were born in a much poorer post-war environment, witnessed the vertiginous rise of Norway’s wealth and benefited from their country’s steady advancement to an affluent, top ranking welfare society (Helliwell et al., 2022; UNDP, 2022). For the older generation, retirement means not only relatively lavish pensions and free healthcare but also opportunity to join a multitude of prosocial networks and initiatives. There are not many societies with whom Norwegian Boomers could compare and not feel uniquely privileged—a predicament likely to inspire happiness and satisfaction.

It is therefore not surprising that the Norwegian Monitor’s data from 2021 show a trend which points to the phenomenon of “blossoming pensioners” (Hellevik and Hellevik, 2021). The plus-60s are now the happiest Norwegians (Figure 1). As happiness among seniors has increased, youth well-being has been steadily declining since the mid-2010s. Data from Statistics Norway (2023) confirm the trend.^2^ Annual surveys of subjective well-being show that, since 2020, individuals aged 18–24 have consistently reported the lowest scores, with each successive age cohort reporting progressively higher levels of well-being. This trend is spreading across Northern Europe: in 13 countries, life satisfaction now increases with age (Blanchflower and Bryson, 2025).

The Norwegian Monitor’s quantitative surveys indicate that young people frequently mention poor social relations, body issues, problems with mental and physical health, economic worries, and a fear for how the future will affect them as individuals. They are increasingly worried about climate change, but the climate concern does not seem to significantly reduce their quality of life. Far more impactful on their well-being is an experience of Norway as a society that it is becoming worse to live in.

Madsen (2021, 2023) questioned whether the decline in well-being may reflect not only changing risk factors, but also evolving cultural frameworks that emphasize introspection, optimization and therapeutic self-understanding. The rise in public concern about youth mental health—often labelled a “crisis”—could contribute to increased pressure on young people, exacerbating their ill-being.

These findings are significant, but they raise several important questions. What are the young generation’s narratives about Norway as a place to live? What gives their lives meaning and a sense of purpose? How do they understand the relationship between their pervasive use of social media and their declining quality of life? To what extent do they feel capable of changing reality for the better? And what factors help explain why many older Norwegians appear to forgo the comforts of a well-deserved *dolce vita* in favor of a “hyperactive” lifestyle?

This article seeks to elucidate in greater depth some of the mechanisms driving the generational shift in well-being in Norway—a country ranked the world’s second-best place to live (UNDP, 2025) and the seventh happiest nation (Helliwell et al., 2025). Objectively, Norwegian youth should feel great about living in the world’s perhaps most successfully run country (Helliwell et al., 2022; UNDP, 2022), not to mention that their material security is much higher compared to most other countries. Why, then, are they so miserable?

### 1.1 Background for our research: Norwegian culture in transition

Diener (2009) emphasized the impact of social values and norms on the well-being of a population. Distinct, value-charged cultural programs inform, at least in part, what societies we build and what ideals we strive for and cherish. Our communities supply the cultural scripts that individuals use to interpret their life situation and experiences of well- and ill-being (Bruner, 1990).

It is nearly impossible to examine the significance of the generational shift in levels of well-being without also considering how this shift relates to the fundamental norms, values, and practices of Norwegian social democracy. Let us therefore briefly outline the main features of the so-called “Norwegian model.”

As we have argued elsewhere (Witoszek and Midttun, 2018), the concept of the Norwegian “well-being society” is not just related to the country’s fossil wealth; rather it is part and parcel of a strong humanist tradition characterized by an emphasis on equality, freedom, welfare, and justice—values which underpin the society’s exceptional prosperity and well-being (Witoszek, 2012; Witoszek and Midttun, 2018; Larsen, 2021, 2022, 2023, 2026). A strong ethos of cooperation and egalitarianism has generated a social democracy in which wealth distribution has been relatively fair and everyone’s material needs are covered. Until recently, the Norwegian Model—marked by a close dialogue between authorities, employers, and employees—has restrained predatory market forces, giving the Norwegians greater income equality and less poverty among vulnerable groups (Fritzell and Lundberg, 2001). A culture of moderation has tempered conspicuous consumption among the well-off. The region’s Lutheran *employment ethic*, as opposed to the American *work ethic*, downplays the importance of economic success (Nelson, 2017; McKowen, 2020). In addition, Norwegians boast a strong nature tradition which is evident both in their pastime habits—frequent hiking trips and cabin visits (Anker, 2022)—and in their self-image as a *frilufts-folk* (“people of the open air”).

In the past decades, this humanist and nature-centered culture has come under siege. The Norwegian Model keeps delivering high prosperity and generous welfare, but neoliberal globalization has influenced indigenous norms and values that have hitherto been anchored in an egalitarian and cooperative ethos. Income inequality has increased, the well-off spend money more conspicuously, and property prices have skyrocketed. Academically ambitious young people struggle with rising grade requirements, which encourage a culture of competition and emphasis on performance and excellence (Pugh, 2015; Kangas and Kvist, 2018; Gustafsson and Palmer, 2019).

In an effort to counter these trends, in the 2010s, the Norwegian authorities moved towards reconceptualizing their vision of a “good society” by emphasizing high quality of life rather than focusing predominantly on economic concerns. This entails a transition from being materialistically-oriented *welfare societies* to becoming more holistic *well-being societies* (Witoszek and Midttun, 2018). Part of the Norwegian governments’ agenda since 2015 has been to promote the ideas of social well-being and develop institutional structures that are meant to help citizens create better lives for themselves.

Ironically, despite this intense political and social focus on happiness—between 2018 and 2025, Norway fell from the top position in the *World Happiness Report* to being number seven. This drop may seem trivial, but it is telling. Has it stemmed, at least in part, from a marked reduction of well-being among the young?

### 1.2 Research objectives and methodology

To answer the last question—and to gain a more nuanced, fuller understanding of the generational shift—our interviews were designed to examine both the “happy” and the “unhappy” generations against the backdrop of a Norwegian model in transition. One objective was to contrast the apparent *tristesse* of Norwegian youth with the remarkable resilience and well-being of seniors—products of an earlier era shaped by social-democratic norms and values. Of course, the teenagers’ experience of anxiety and frustration does not necessarily mean that they lack resilience (Rybak and Szmyd, 2025); rather it invites to identify manifold psychological, economic, cultural—even narrative—mainsprings of their tristesse. Hence our aim was to identify the key factors—expressed either explicitly or implicitly by young people—that contribute to their experiences of ill-being. In particular, we sought to explore how younger and older Norwegians interpret their rapidly evolving, digitally driven life-worlds. Finally, based on a close reading of the interviews, we attempted to reconstruct the aspirations and values central to both generations and to relate these to the norms and ideals long embedded in Norwegian culture (Witoszek, 2012; Anker, 2022). Although our findings remain tentative, they serve as a potential point of departure for a broader investigation into the relationship between human well-being and the ongoing crisis of democracy.

Our objectives weighed on the choice of methodology. To deepen our understanding of the multiple factors that promote or hinder well-being across generations, we conducted a series of qualitative interviews using a triangulation strategy (Denzin, 1978, 2017; Patton, 1999) that combined focus groups, individual interviews, and conversations with experts such as social workers, teachers, and health professionals. In addition, to identify the often opaque and underexplored sources of ill-being—and to corroborate our interview findings—we reviewed selected public debates in the Norwegian press concerning the decline in youth mental health. In addition, through searches conducted in the database Retriever—encompassing newspapers, online platforms, and academic journals—we selected roughly 35 articles, commentaries, opinion pieces, and letters addressing youth, mental health, and related issues from the years 2023–2025.

Our choice of multiple informants—as well as several information platforms—justified the need for a *multiperspectival approach*—one that captures the complexity of well-being rather than reducing it to a simplistic “happy–unhappy” dichotomy (Larkin et al., 2019; Hocking and Crichton, 2024). By drawing on diverse viewpoints and comparing different perspectives, experiences, and interpretive frameworks, we approached well-being as a dynamic process in which different stakeholders—and meaning-makers—illuminate distinct, and often obscured, facets of national health and well-being which are not captured by opinion polls.

Methodologically, our interviews were also guided by a narrative approach, focused on stories, tropes and images used by our informants to express their predicament and imbue it with meaning. Bruner (1990), the pioneer of a humanist-oriented “folk psychology,” argues that humans are “meaning-making creatures” who interpret and navigate their lives primarily through culturally shared narratives. It is these meaning making stories that we tell ourselves—and the outside world—that give us wings and open our minds, or make us despondent and resigned. While talking to our old and young informants, we focused on narratives, tropes, and images that they used while describing their predicament. A combination of narrative and multiperspectival approach allowed us to better grasp diverse sources of generational happiness switch, filtering it through different points of view, standpoints—and different set of value-charged stories and images—rather than relying on only one explanatory frame.

## 2 Research design: selection of informants, guiding topics, and research questions

We arranged individual interviews with focus groups including high school students (*n* = 19) from four schools: one in a prosperous part of Oslo, another downtown, and two in small towns on the periphery the capital. One group comprised vocational school students, while the others included more academically oriented teenagers. Several of our student informants had an immigrant background.

We made sure that our older informants also represented a broad social spread. One focus group included seniors from the prosperous, western part of Oslo, another from traditionally lower-middle class districts (*n* = 12). We also conducted in-depth individual interviews with experts, such as teachers, union representatives, social workers, psychologists, an educational researcher, and a priest (*n* = 17) (Table 1). To corroborate findings stemming from our interviews, we reviewed a selection of national debates on the teenagers’ mental health in the Norwegian mainstream press in the period 2023–2025.

**Table 1:** Informants.

High school students	8 females (aged 17–18), 11 males (17–18)
Seniors	4 females (68, 71, 73, 78), 8 males (67, 72, 73, 74, 75, 75, 78, 80)
Experts	Male priest (74), male teacher (40s), male former teacher (72), female union representative (62), male head of school leader organization (62), male former teacher and educational researcher (67), female psychiatrist (65), male psychologist (56), female psychologist (63), female student well-being advisor, female clinical psychologist and psychoanalyst (42), female leader of a mental health organization (59), male specialist child and adolescent psychologist (65), female media studies scholar (43), female clinical child and adolescent psychiatrist, male youth and identity psychologist, male psychologist and philosopher (45)

We recruited students through contacting school administrators who had teachers ask for volunteers for interviews on youth’s well-being. Seniors were recruited via personal contacts, and a snowballing method. Our informants were selected through purposive sampling (Schreier, 2018), as a result of personal contacts, online searches, or media appearances. All our informants were either born—or were long-term residents—in Norway. Our inclusion criteria were based on establishing whether the informants had relevant personal experience related to our theme, or a particular expertise in the field.

Our semi-structured interviews were inspired by the Socratic method (or dialectical inquiry), designed as dialogical exchanges that pose similar questions in different ways to uncover underlying tensions in participants’ responses. To capture both the *mindscape* and *taskscape* of our informants (Ingold, 1993)—that is, their perceptions, narratives, and practices—we discussed the following themes:

1. Perceptions of Norway as an ‘exemplary democracy’
2. Relationship with smartphones and use of social media
3. Experiences of social life
4. Participation in voluntary organizations and contributions to the public good
5. Use of nature as a space for renewal
6. Ideals of a “good life”

Our interviews were semi-structured, meaning that they took the form of a conversation where we allowed our informants a certain degree of freedom to stray away from answering the guiding question—or not answering them at all. This ploy followed Lotman’s (1990) semiotic point: “What is said is equally important as what is not said.” The key questions which were the basis for a conversation included: How do you perceive Norway as your ‘home’? Have things got better or worse in your country in the past decade? Do you trust your government and Norwegian public institutions? To what extent do you agree with research suggesting that, for the first time, Norwegian youth are less happy than their parents? If you agree, why? If not, why not? Can you describe your relationship with your smart phone? How dependent you are on social media? How often do you spend time in nature, and if not, why not? What is your idea of a ‘good life,’ and how do you imagine yourself 20 years from now?

Our interviews—all of which were face-to-face—followed a scenario where one of us led a conversation, and the other kept taking notes, occasionally intervening and asking informants for clarification. The interviews were recorded, then transcribed with software and quality-proofed manually, done by the article authors and a research assistant. Direct quotes have been slightly edited for readability. A majority of our informants opted for anonymity.^3^ We employed a grounded theory approach (Glaser and Strauss, 2017), which entailed an interplay between data collection and analysis through the interview period November 2022–May 2024. All respondents were provided with forms and asked to give informed written consent. We stopped recruiting informants when reaching saturation in terms of novel information per interview. Ethics approval was obtained in line with the Norwegian decentralized model. Our project was assessed by the Norwegian Agency for Shared Services in Education and Research (reference number 124504).

## 3 Results

### 3.1 “Something rotten” in the state of Norway?

With the exception of students from vocational schools, most young informants questioned the notion of Norway as an exemplary democracy. “There are too many things that do not work—like the long waiting time to see a doctor,” said Maria. When asked to elaborate on their ailments, many referred to anxiety and stress, and to their need for psychological support. Several expressed distrust in public institutions. “What politicians say and what they do—there’s a big gap there,” said Arne. “And I do not think politicians care about us or our mental health.”

“On the whole, things are getting worse every year. Look at property prices—they are insane,” said Arne. While interpreting and discussing the typescript of our young informants’ comments, we noted two regular discursive ploys. Firstly, irrespective of the topic (cost of housing, perception of school system, performance pressure, etc.), coruscating in the teenagers’ answers were unaddressed mental health issues springing from the youth’s anxiety and fear. Eivind did not think that his school did a good enough job to tackle his experience of loneliness and despondency: “If you are having a tough day, it’s hard to find someone to talk to. The school employees who *should be responsible* do not know how to deal with such things” (our italics). Samira said: “It is hard to ask friends…. Many people are lonely now, much due to social media…They talk a lot about mental health at school, but they do not do anything.”

Blaming the state—and state institutions—as responsible for one’s mental problems is one of the paradoxes of the Norwegian model, whose young inhabitants are both individualist and demand autonomy, but—when in trouble—are programmed to take refuge in the welfare society and blame state institutions for their predicament. That said, one of the interviewed teachers insisted that the institutional focus on mental health has gone too far: “Schools talk too much about mental health. Influencers too… This, in combination with how the welfare state promises too much, makes many young people expect a life of happiness, ease, and comfort. They do not learn that life will be hard and that they will have to work hard too.”

In sum, while describing Norway as a place to live, most students spoke about a country where both their material and mental well-being were fragile and under strain. Interestingly, in their rhetorical depictions of Norway as a place, many students described their country in psychological terms—not as a habitat, but as a locus of a negative state of mind marked by pressure, loneliness and frustration, with little or no healing in sight.

### 3.2 Youth ill-being and social media

Most of our young informants believed that social media has contributed greatly to radical changes in their lives. There was an interesting rhythm in our conversations. At the onset, most teenagers struggled to offer a balanced picture of the social media’s role in their lives. The high school students alluded to many positive sides of their smart-phone-directed routines: being part of the community, staying informed, and getting friends. But as we continued our conversation and asked for elaborations, they switched from the balanced to an increasingly negative modus. Many alluded to feeling “powerless” and “overwhelmed” by the excess of largely negative information flowing from their smart phones. Some found themselves trapped in a downward spiral mirroring effects of addiction. As Nikolaos (18)^4^ put it, “My older brother warned me that social media would make things worse for me than for him, and even worse for our younger sister. TikTok destroys their brains, the worse the younger they are.” Mateusz (17) added, “Your brain, motivation—everything is affected.” Trym (17) said, “Kids are so young when they begin watching TikTok where the content can be very adult. They get strange expectations and grow up faster… They stop playing with friends. The streets are empty of teens now. They are home, on the computer.”

Hate, fear, negativity, boredom, addiction, bullshit, gloom, and feeling bad, restless, lonely, useless, and distracted were the most common words used by our young informants to describe their relationship with their smart phones. “I can no longer watch long videos on YouTube because I get bored,” said Louise, “When I do school work, I constantly have to pick up the phone to watch more TikTok.” Samira had a similar comment: “I speed up videos on YouTube. People speak too slowly. TikTok just distracts me, I spend so much time there, but get no use from it.” Cecilia concurred: “TikTok could make worse an already gloomy day. Their algorithms pick up that you are feeling bad. Then they give you a lot of dark content, dragging you further down.”

Many students declared that they social media blurred the distinction between rest and activity. “Peace and quiet get very boring. I cannot think for more than 2 min. I have to pick up my phone to get stimulated because I’ve had it since I was so young.” Sven (17) signaled the same problem: “I do not know what to do without the phone in my hand. I only let go when I go to sleep.” Nikolaos declared: “Do not you see, we all have ADHD now, so we must have non-stop action.”

Most students agreed that, as a consequence of their addiction to TikTok, Instagram, and other platforms, their interpersonal relations “were a mess.” Nikolaos said, “Kids do not get along anymore. Everyone now wants to be the top shot, like wannabe gangsters.” When asked to clarify how the social medias’ images of the “gangster Übermensch”—especially prevalent about male teenagers—affected their social relations, some students alluded to an increase in aggressive behavior. “People just threaten each other,” said Mateusz. “You may have friends and all, but you are not safe.” Sven concurred: “The new media have made people turn on each other. Because so many are bored and angry, they use social media to [get their frustration out], to make trouble for others. There are always threats being thrown around online.” Mateusz added “You often get tons of hate when you are gaming. People do not go around threatening each other face to face. It’s easier over the internet.” Things felt dramatic by Adam who insisted that he no longer could help the feeling that someone walking behind him had evil intentions: “He might want to stab me.”

### 3.3 Comparison is the thief of joy

Students at the westside Oslo school emphasized the ways in which social media ‘forced’ them to constantly compare themselves to others. One said, “[On screens] we only see the good sides, which can be fake. And you only get to see the impressive result, not the hard work behind it.” Most students felt pressured to look better, talk faster, and to have a more enviable love and family life. “You see guys with sixpacks all over social media, but in reality, very few guys are like that. It hurts when you fail to live up to these ideals,” Samira said, “When insecure people see prettier people online, they cannot help making comparisons. Then they self-diagnose with anxiety.” The compulsion to compare oneself with others made the ideal of happiness not only desirable but imperative: “We must have money, spend plenty of time with family, and be perfect in everything.”

Many students felt that the comparison trap forced them to constantly share glimpses of their “happy life” with others. “My family always takes photos, joking that if it’s not documented, it did not happen,” said Trude. Everyone in the focus group agreed that if they failed to share a good experience on social media, it was worth less. “We are a sad generation with happy photos,” said Anne.

One source of the teenagers’ grief was comparing their own constrained world with the happy-go lucky life of their grandparents. “We have been told that we are a sadder generation, but this cannot be only because of social media,” said Cecilia, “We worry about the future. Previous generations had it easier. So many professions are gone, and you need crazy-high grades. People used to just study law if they wanted to, to be what they wanted to be. I always wanted to be a nurse, but I had to give it up because nurses are paid too little. What if I became a single parent? As a nurse, I could not afford to live in Oslo.”

Dagne (62), a former educator, agreed: “There is a much stronger focus now on what to become and what grades students must earn to get to the top. Instead of figuring things out as they go along, they have to plan.” Stig (62), a former principal who leads a school leader organization, concurred: “At my school, we used to have a nurse on standby when exam prompts were read out. Students were passing out. Grade requirements are so high now that parents take days off from work to engage in their children’s exam preparations. Not to mention that there are so many arenas they must perform in. They must make TikTok videos, work out, be social, do homework, and then reply to 50–60 messages on Snapchat. Maybe being so busy looks good from the outside, but what about how do they feel on the inside?” Børge (67), a former teacher and educational researcher, has noted that the hyper-active youth is less affected by the social media and more by an incursion of a neoliberal values into Norwegian education: “High schools are no longer about *Bildung,* but about market thinking. Education has become a service to help students get the best-paid jobs.”

### 3.4 Digital stigmatization

What was clear in our informants’ comments was the ambivalence social media as a double-edged sword: on the one hand the source of virtual community and belonging, on the other a “new drug” creating a sense of disempowerment and “willing” loss of freedom. Being dependent on gadgets should have made the world expand but instead it shrank the mind and soul. But there was one more, implicit story which hid in students’ comments, one which tangentially related to the stress about creating an attractive digital self. Buried in the students’ complaints was the fear of exclusion from a digital community. Psychologists talk about a FOMO effect: the fear of missing out (Herman, 2000; Zhang et al., 2023). But we noted that there is more to FOMO than the fear of not being *a jour* with what is happening, overlooking parties, and missing social events. There is a fear of rejection, of being stigmatized as a “freak” or “weirdo,” as “not one of us.”

Goffman (1963) defined stigma as “an attribute that is deeply discrediting,” which reduces a person “from a whole and usual individual to a tainted, discounted one.” Though none of our young informants mentioned the word “stigma,” many referred to a fear of “being marked as flawed,” feeling mismatched with the rest of their peers, not being part of the digital community. As one teenager put it, “We have this one guy in class who does not use social media. I do not know why [he made] such a huge choice, so much happens in there—it’s a bit scary.”

Digital stigmatization is a relatively new phenomenon, not specific to Norway but increasingly widespread across the virtual world. The victims of digital stigma are made to feel less attractive or less sexy, or are labelled as strange, “freaky,” or “fake.” As one of the interviewed psychiatrists put it: “Many young people come to me with conditions for which we have no designations—no established names in standard medical discourse. One of these is becoming a victim of digital stigmatization: becoming a virtual outcast, a victim of digital rejection because one does not fit in, is too fat, too ugly, or even too smart. How are we to name this predicament? Is it a medical condition—and if so, what is the cure? Sometimes it produces a mixture of psychosis and anxiety, but these terms are far from adequate.”

Being excluded from a community—digital or otherwise—is a serious predicament that entails humiliation and a profound lack of belonging. Our psychiatrist explained: “Cognitive behavioral therapy can help somewhat by addressing negative thought patterns, building coping skills, and treating depression often linked to exclusion. But we doubt that it can fully address digitally induced stigma, “belonginglessness,” and their emotional consequences. My suspicion is that, by analogy with couples therapy, we may ultimately need a form of **“**perpetrator–victim therapy” to address the relational dynamics at the heart of digital exclusion.”

### 3.5 Happy plumbers and electricians?

One of our discoveries in the process of interviewing teenagers has been that the social media—and the pressure on achievement, excellence, and performance—seem to have a negligible impact on students at vocational schools. Students who choose—or who are put in—these schools have a dual status. On the one hand—associated with the “laboring classes”—they are perceived as “left-behinds” or as lacking ambition. Vocational schools train them in “grounded” work, solving practical rather than abstract problems, and attention to concrete, often demanding tasks which do not allow for TikTok-ing or Instagram-ing during work. Pupils from vocational schools only need passing grades to move on to becoming professionals. On the other hand, in Nordic countries, electricians and plumbers are usually paid more generously than in countries with greater income inequality.

In our vocational school focus group, there were no students who complained about peer pressures, anxiety, ADHD, and other digitally induced ailments. Everyone felt certain that they would soon get jobs that paid enough to secure the lifestyle they wanted. Unlike their academically oriented peers—who desired success and fame to get their “name out there”—these vocationally-minded students seemed rather relaxed, while their dreams were strikingly pedestrian. Geir stated: “I want to be independent, make enough money, have a job, and a nice, stable life with a good friend group.” “I do not need any luxuries.” Nikolaos added, “I want to have an apartment before I turn 20, a car by 21. Have a wife and 2 healthy children before I turn 23. No stress, relax, enjoy my family—that’s life.” Terje wanted a bit more, “A nice car would be cool if I could afford it, but no worries about that. I do not need that much to be happy. Just a girlfriend, 2 kids, a house, and stability.”

The Norwegian Monitor’s surveys show a significant correlation between materialism and low subjective well-being. Hellevik (2015) found that Norwegian materialists feel that, in order to live a good life, they need an income that is 35% higher compared to what their idealistic counterparts report. The conclusion has been that contemporary youth “are more preoccupied with consumption and acquisition than older generations” (Hellevik, 2015). The vocational students’ conceptions of well-being challenge some of these quantitative findings. The “good life” envisioned by aspiring plumbers and construction workers is not defined by lavish excess—a Ferrari outside a palatial villa or a yacht in the Oslofjord—but by the security of steady work and the simple, everyday pleasures of a happy family life, rather than the temptations of conspicuous consumption.

### 3.6 Nature, voluntarism, and civic engagement

Addiction to smart phones and an increasing performance pressure seem to draw the young away from outdoor life (*friluftsliv*), which has been an enduring source of Norwegian identity and well-being (Witoszek, 2012; Anker, 2022). Only a few high school students appreciated hiking and cabin life, and none to the extent that earlier generations typically did. One said, “I enjoy being in nature, but when I am there, I see on social media how my friends are partying in the city, which makes everything else seem boring.” Like several of our informants, when Cecilia is in nature, she listens to music on a headset: “I cannot stand the peace in nature. It’s too quiet.” The students rarely use nature for exercise. They prefer to go to the gym, where energetic training gives faster and more visible results. A teacher thought this development was unfortunate: “Screens weaken our connection to nature, which is sad because nature connects us to something besides ourselves, a connection that helps us land a bit.” He also saw negative aspects to how gyms draw people away from team sports: “When students go to the gym alone, they get stronger, but they are not part of a team striving to reach goals together. Togetherness helps us transcend egotism.” Trond (74), a retired priest, made a similar point: “Happiness is found in a community.”

Social media seem to have eroded another practice that has been one of the pillars of Norwegian identity: helping those in need. Compared to their grandparents, our young interviewees had a strikingly indifferent relationship to work for the common good. Traditionally, Norway has boasted world-leading levels of volunteering (Plagnol and Huppert, 2010), which can increase the well-being of both helpers and those being helped. Voluntarism builds social trust, too, a central component of Nordic culture (Larsen and Witoszek, 2023). None of the students at the westside high school had a track record in volunteering. The students who were socially engaged did so mostly to get personal benefits, such as free access to a festival. The vocational school students chuckled when asked if they engaged in any voluntary work. Terje said, “I only help if I get money, or I will be taken advantage of. People have asked for my grandfather’s help without giving anything back, which is abuse.” Sven was brutally honest: “[Helping those in need?] I have other things to think about.”

Several students worried about climate change, but most expressed a nothing-can-be-done attitude. One said, “I’ve given up on fixing the climate. I admire those who fight for a better world, but I see little change. It’s not worth the sacrifice of getting engaged. See how much Greta Thunberg does, and not even that helps.” Another added, “Everything is about money in this world—that’s the bad part. The world will only be saved if businesses can make money doing it.” Climate activism was sneered at. “My only social engagement,” said Arne, “is to challenge other people’s opinions online.” Trym agreed: “Only girls care about the climate. We are busy every day, so we do not have time. It’s for those who are bored.” “I used to care about the climate,” added Terje, “but now, I’ve given up. Politicians only talk, and little happens. The next generation will have to fix it, but it’s impossible.”

To sum up: civic engagement was not the young generation’s priority. Some of our young informants indicated that contributing to the well-being of others was thinkable only after they themself had flourished as individuals. Their focus on the self was strongly related to the ideals of individual freedom and independence. Cecilia said, “My parents gave up their freedom to have children. I have to set my own freedoms first, since I am the one who has to live my life.” Geir tied freedom to money: “When everything is as expensive as it is now, this gives you less freedom. To enjoy friends, to go out and eat, transport, drinks—it all costs money.” He, like the others in his focus group, stated that they do not care about politics: “It’s like the climate. Politicians talk instead of doing. I do not watch the news.” Trym added, “When we turn 18, we probably will not vote for another 5 years.” Geir said, “We do not pay attention, so we do not know who to vote for.”

The students seemed to recognize that their political detachment aligned poorly with core Norwegian values, which emphasize strong civic engagement. Their lack of social involvement, however, did not appear to trouble them. As one teacher concluded, “Social media can make you care less about others, about fellowship, and about politics. They weaken democracy.”

### 3.7 The happy grandparents: the puzzle of senior well-being

As noted above, over the past decade—and in stark contrast to the anxious younger generation—many Norwegian pensioners have continued to enjoy high levels of well-being. Many now recognize the exceptional stability they have experienced, which accounts for their unshakeable image of Norway as being an “exemplary democracy” and “one of the world’s best countries.” When pressed, they admitted that increasing individualism, self-centeredness, and polarization undermined social trust. Trond said, “Our society is to an exceptional extent based on trust. We still have trust, but less and less so. Equality is the prerequisite for high social trust. Without it, we become more like South Africa where everybody hides behind walls, afraid to be victimized by those who suffer deprivations.” Most of the seniors thought that the negative development was bound continue in the years ahead. But only one, John, dared to play a Cassandra. “The Nordic Model is disintegrating from below,” he declared, while the others smiled with kind disbelief.

As Trond explained, “The oldest [of us] have lived through [Norway’s] golden age. Most of us have had uncomplicated lives where everything just got better. The young have to live with the danger signals: climate change, war, and harder lives with more demands.” John (78) concluded, “Ours is a very privileged generation, perhaps uniquely so. All our positive expectations have been fulfilled. This can no longer be taken for granted.” Merete agreed: “We are done chasing careers. We’re satisfied with our lives. If someone makes more money, or has a nicer house, it does not matter.” Eva added: “Young people are going places, figuring out who they are. We pensioners have landed, we have good pensions, and we have become more secure about who we are. I feel deep gratitude for having lived such a good and meaningful life.”

Most of the seniors we interviewed (aged 67–80) stated that they were in good mental and physical health. In a conversation they referred rather discretely to “standard age ailments” (physical disabilities or losing a spouse), but they were not eager to expand on their health issues. Anna laughed off the question: “we have good medical treatment, active outdoor life, and do voluntary work, such as helping integrate immigrants, so we do not have time to think about our pains and aches.” Thorvald (75), beaming with enthusiasm and *elan vitale,* explained, “I feel like we almost have a duty to contribute. I take it upon myself to help others, also because it gives me joy, perhaps not always in the present, but knowing that I am useful to others gives me self-esteem and self-respect. Helping others is not quite an obsession, but certainly a top-level priority in my life.”

Pål (74) had found it challenging to retire: “Being raised in the Protestant tradition, I had internalized that you have to contribute before you consume. I had to reorganize my life so that I could keep contributing to others.” While engaging in voluntary organizations helped him maintain well-being, he has also begun worrying about the world which, according to him, is on a slippery slope: “Poverty. Mental health among the young. How climate change will affect my children and grandchildren. War. Migration. Increasing inequalities.” But then he concluded: “Yet I am unable to fully absorb the reality of these challenges, so I sleep well.”

Those with grandchildren derived well-being from helping relieve stressed parents, yet they too were worried about the future of their offspring. John declared, “I have no fear of any threats to my well-being in the years I have left. But I feel bad for my children and grandchildren, although such worries reduce my own quality of life far too little. These are such large, existential problems that I am unable to relate to them on a day-to-day basis.”

The seniors spoke nostalgically of their creative childhood. They rekindled memories of playing in the street, inventing games, the challenge making something out of nothing. Solitsky (80) said, “We experienced unexpected situations and dealt with them then and there. We did not write SMSs to each other, we talked. We made toys from what we found [in our surroundings] and we played happily without any gadgets. Today kids do not even know how to play hide and seek. They are helpless and bored without a smart phone or a tablet. They expect to be entertained, not to entertain themselves. But happiness is the sum of what you have experienced, so you cannot delegate your own happiness to others. You must experience the negatives to get the highs.”

Many of the older informants continued the *friluftsliv* habits of recharging their batteries and “healing their souls” through activities in nature. They all confirmed that communing with the natural environment was one of the main sources of well-being. Hiking in the mountains, fishing, skiing, and spending time in their cabins—with the accompanying maintenance work—filled large portions of their time and induced meaning into their lives. Generous pensions allowed them to take long vacations either at home or in exotic places which enhanced their quality of life.

Most seniors we interviewed mentioned that they stayed away from Facebook and other social media. Some made a point of only using emails for group communication, even if they found it cumbersome. Those who used social media to stay in touch with friends, tried to do so in moderation and without the need to compare themselves to others. One said, “We do not care about comparison. We are done chasing careers. We’re satisfied with our lives. If someone makes more money, or has a nicer house, it does not matter.” Eva added: “Young people are going places, figuring out who they are. We pensioners have landed, and I just have to be satisfied with who I am.”

### 3.8 TikTok and other bogeys: insights from media debates, health experts, social workers, and teachers

The phenomenon on the Norwegian “anxious generation”—to allude to the title of Haidt’s (2024) influential bestseller—has been the focus of an intense national debate in the Norwegian mainstream press. In line with our multiperspectival approach—and to illuminate the equation: toxic social media = poor mental health = decreased well-being—we also interviewed health experts, teachers, and researchers. Both the media debate and interviews point to a marked disagreement among the health workers and educators on the causes of the decline in “gross youth happiness.”

An illustrative example comes from 2023, when Ringdal (2023), a lecturer at Kristiania University College, asked his students to write about something they cared deeply about. In a debate piece published in the Norwegian online newspaper Khrono, he wrote that he expected his students to write “essays on politics, careers, technology, football, love, or climate change.” Instead, over 90 percent of the assignments were about mental health struggles, anxiety, and depression. Student after student described fear, self-loathing, and inner turmoil. Several wrote about obsessive thoughts and loneliness. One wrote, “I had to talk to someone. The chaos inside me paralyzed me. I felt dizzy. It was so unusual to feel heard”.

Ringdal (2023) summarized: “When I was in my early twenties, we discussed nuclear weapons, war, capitalism, climate change, and love. We looked outward—at power, at society, at the world. The new generation seems to look inward. Not because they are self-absorbed, but because that is where the pain and fear reside. Young people today live in a constant stream of evaluation and reflection—a perpetual fear of being seen, judged, or exposed as less successful”.

Similarly, a study published in *The Lancet* by a Scandinavian research team found that one in three students reported mental health disorders (Sivertsen et al., 2023). For women, the number was nearly 40 percent. Some Norwegian psychologists recently sounded the alarm. In an interview published by the academic debate outlet *Khrono*, Svein Øverland stated that “so many young people are now broken [that] we are heading straight for disaster, and it’s already too late to turn things around” (Hystad, 2025).

Yet Norwegian researchers and experts are divided on the question of an allegedly “broken” generation. In an opinion piece in the major Norwegian daily *Aftenposten*, psychologists Ole Jacob Madsen and Djup (2023) offer a sobering perspective on seemingly unhappy youth: “We live in a time where psychology, introspection, and the staging of psychological struggles have taken center stage.” While self-reported mental health issues have increased, “it is an exaggeration to claim that students are uniquely vulnerable.” Madsen and Djup (2023) see at least two hazards related to the national obsession with psychic ailments. First, the promotion of openness about mental health carries the risk of “romanticizing suffering, medicalizing normal life struggles, and cultivating a victim identity.” The second hazard has to do with the narratives of an unhappy youth tending to become a “self-fulfilling prophecy which places additional strain on mental health services, neglecting patients who truly need help.”

Our interviews with health experts and teachers shed additional light on the drivers of youth’s unhappiness and nuanced our findings. To summarize the most striking arguments: Some teachers referred to mental health problems as related to what we call a suppressed cultural shock. The shock in question has to do with Norway’s transition from a cooperative to competitive society. While teamwork and prosociality are the Norwegian cultural DNA which still dominates in early childhood upbringing, adolescence marks a transition to a competitive modus, which, when exacerbated by social media, may lead to traumas. The individualist ethos, which emphasizes self-optimization, adds to an ongoing stress, and erodes collective action which is the fundament of Norwegian social democracy. Social workers, on the other hand, identified family breakdown as one of the sources of growing unhappiness. Divorce, which is almost a norm in Norwegian urban settings, leads to the youth having to shift between families and navigate between different homes—a cause of anxiety and uncertainty about the future. A number of psychologists and health experts referred to a crisis of masculinity: boys are losing status in a school system where “alpha girls” get top grades. Interestingly, boys are more often diagnosed with biological conditions (ADHD, autism) which require invasive pharmacological intervention, while girls’ problems tend to be framed in terms of disruption in social relations, and hence reparable via psychological counselling. This differentiation intensifies the disquieting self-image of young men as “sick” or as “losers,” in contrast to girls whose problems can be solved without chemical side effects.

Some expert informants talked about youths’ psychological problems as related to Norway becoming a multicultural society where, increasingly, non-Norwegian boys and girls suffer from split identities. Many struggle to satisfy colliding sets of norms and values: one coded in traditional family imperatives, the other valuing autonomy and emancipation—a process which increases stress and anxiety in ethnic minority students.

There was one more, largely under-researched problem behind the phenomenon of “unhappy youth.” One teacher informant called it a **“**corporatization of schools.” Schools are increasingly treated at business corporations, with parents and pupils—reduced to “clients”—enter into a transaction with school principals and teachers. The Norwegian school that has lost its humanist fundament and replaced it with economic logic, generates disgruntled consumers rather than responsible citizens. The question to what extent the teens’ declining mental health has a tacit connection with redefining schools as market arenas needs more research. Certainly, the corporatization of schools goes beyond the encroachment of rivalry and competition in young generation’s lives. Part of the pervasive commercialization of the schooling system has been over-diagnosing of the youth’s health problems. According to our teacher informant, “The welfare state’s fund-chasing strategy means that the healthcare system—always in need of resources—has an incentive to formally diagnose pupils as mentally ill or unstable.”

Intriguingly, two of our teacher informants mentioned the crisis of the teachers’ authority as an indirect source of student unhappiness. Over the last decades, Norwegian schools witnessed not just an increase in student bullying, but a wave violence and harassment directed at teachers. In their stories about their traumas (Skåland, 2016; Lindh, 2022), some teacher informants referred to schools as a “theatre of cruelty.” They cited countless violent incidents against school staff ranging from spitting, kicking, attempts at strangulation, to assaulting pregnant teachers.^5^ According to some teachers, the progressive demotion—and humiliation—of teachers leads to a massive exodus of gifted educators from teaching positions, thus lowering standards in Norwegian schools. The progressive deterioration of teachers’ status as guides and role models leaves youth confused, disoriented, and becoming easy prey to a stream of influencers’ demagoguery flowing from the social media.

## 4 Discussion

Our project of studying the unhappy youth and happy seniors in one of the world’s happiest countries is certainly a work in progress. In our questions to the old and young Norwegians, we have focused on narratives which depict Norway as a place to live, express the experience of digitalized world, construe social relations, and portray the ways the old and young use traditional emblems of identity: voluntary work and *friluftsliv*. We are aware that the limited number of our informants offers only partial answers. Our qualitative approach, however—based on narrative psychology (Bruner, 1990; Vassilieva, 2016), cross-checking multiple sources and informants (Denzin, 2017; Hocking and Crichton, 2024)—have helped us both problematize quantitative findings and nuance standard perceptions of the seemingly nefarious role of the social media as key generators of unhappiness among the youth (Turkle, 2015; Haidt, 2024).

Our interviews with high school pupils revealed not only the young generation’s love-hate relationship with the social media, but uncovered pivotal contradictions inherent in the narrative about the “caring welfare state.” Critical as they were of their inability to put away their smart phone, many young informants tended to blame outside actors—teachers, social workers, or the Norwegian health care system—for their addiction. Certainly, more research is needed on the consequences of the public perception of the “welfare state as a Santa Claus” that is expected to make all citizens happy and take responsibility for their health problems, and at the same time respect individual autonomy, including freedom to use the social media *ad libitum*.

Our senior informants—having only adapted to social media late in life—anchored their well-being in cherishing their family and friends, voluntary work, and in regenerative communing with fjords, forests, and mountains. The tradition of Norwegian interaction with nature seems to have lost much of its appeal to the young—at least those living in the city. Hiking or going to a cottage is now surveyed through the eye of Instagram and distracted by a stream of virtual messages. It is too early to estimate the impact of the “nature deficiency disorder” among the Norwegian youth (Louv, 2008). Again, the subject needs more extensive research, but our findings suggest that there is a half-suppressed *friluftslivs crisis* among the young generation, a cultural anomaly which calls for more studies on the relationship between young people’s alienation from nature and their general ill-being.

Our interviews have also showed how the distinctive Norwegian cultural context—especially the founding narratives and practices promoting prosociality and cooperation—collide with the competitive mindscape promoted by the social media. As we have argued, the strong competitive ethos is a relatively recent cultural import in an egalitarian country like Norway, and so is the perception of Norwegians as “underachievers.” An interesting exception among the generally unhappy youth were our vocational school interviewees, who—confident of getting relatively well paid and meaningful jobs—were both more critical of the social media and seemed happier than their academically-minded peers. Perhaps the way to well-being lies in combining digitally run curricula with carpentry, gardening, and other practical activities that demand maximal focus and temper the digital addiction?

It is too early to predict whether the general loss of well-being among the young is a passing or more enduring trend. It is equally difficult to foresee whether the narcissistic, social media driven focus on the self— signaled by many of our young informants—will persist.^6^ Were we to extrapolate from our interviews, the pivotal values of Norwegian social democracy are under stress in the digital age. The negative correlation between digital addiction and decline of democratic institutions has been pointed to, among others, by Jonathan Haidt (2024, 2025). According to Haidt (2022), “Social scientists have identified at least three major forces that collectively bind together successful democracies: social capital (extensive social networks with high levels of trust), strong institutions, and shared stories. Social media has weakened all three.” Similarly, Lorenz-Spreen et al. (2023) argue that there may be a salient connection between widespread use of social media and such phenomena as growing populism, acute social polarization, and plummeting public trust in democratic institutions.

As our interviews revealed, there is a largely overlooked, potential crisis that has to do with young people’s unhappiness and the future health of Norwegian democracy. This crisis stems from a schooling system that places ever-increasing demands on teachers while simultaneously undervaluing their role as moral guides and champions of democratic values. When teachers lose authority, dignity, and security, the school’s capacity to nurture free-thinking, resilient citizens is seriously compromised. We are only partly ironic by concluding that, in this context, the older generation of happy, healthy—and socially engaged—pensioners may have an important, and so far understudied, role to play in helping renew and strengthen Norwegian democracy.

## Data Availability

The original contributions presented in the study are included in the article/supplementary material, further inquiries can be directed to the corresponding author.

## Footnotes

1 Hill and Buss (2008) note how one finding seems to contradict that happiness derives from solving adaptively relevant challenges: how having children reduces marital happiness. They suggest several possible proximate causes to this conundrum, for instance, that modern couples lack a large network of extended kin who could help out with offspring.

2 Statistics Norway assesses subjective well-being through 12 indicators that cover satisfaction, optimism, meaning, engagement, mastery, social relations, and positive emotions.

3 We do not specify which names are pseudonyms. To preserve the privacy of the anonymous informants, interviews are not publicly available.

4 All given ages refer to the time of the interview.

5 Some insights from our interviews with teachers were published in an op-ed (Witoszek and Larsen, 2023).

6 One of our commentators suggested that perhaps all young generations are somewhat narcissistic, but then get socialized and “grow out of it.” Social media may boost and prolong the self-centered phases in young people’s life.
